# Current understanding of nonsurgical interventions for refractory differentiated thyroid cancer: a systematic review

**DOI:** 10.2144/fsoa-2021-0041

**Published:** 2021-06-15

**Authors:** Heidi Jones, Victoria Green, James England, John Greenman

**Affiliations:** 1 Department of ENT, Hull University Teaching Hospitals NHS Trust, HU16 5JQ, Hull, UK; 2 Department of Biomedical Sciences, Faculty of Health Sciences, University of Hull, HU6 7RX, Hull, UK

**Keywords:** chimeric antigen receptor T-cell therapy, immunotherapy, oncolytic viruses, secondary therapy, thyroid cancer, tyrosine kinase inhibitors

## Abstract

Thyroid cancer incidence and related mortality is increasing year-on-year, and although treatment for early disease with surgery and radioiodine results in a 98% 5-year survival rate, recurrence and treatment refractory disease is evident in an unacceptable number of patients. Alternative treatment regimens have therefore been sought in the form of tyrosine kinase inhibitors, immunotherapy, vaccines, chimeric antigen receptor T-cell therapy and oncolytic viruses. The current review aims to consolidate knowledge and highlight the latest clinical trials using secondary therapies in thyroid cancer treatment, focusing on both *in vitro* and *in vivo* studies, which have investigated therapies other than radioiodine.

## Lay abstract

The rates of thyroid cancer and related deaths are increasing. Differentiated thyroid cancer is the most common type of thyroid cancer. Early disease can be treated with surgery and radioactive iodine with very good outcomes. However, this therapy does not work for a small number of patients making it important to find different (secondary) treatment options. This review summarizes the results of published research about secondary treatment options for differentiated thyroid cancer. Ongoing research including laboratory-based and clinical trials are also highlighted.

Thyroid cancer is the most common endocrine malignancy [1] and the 20th most common cancer in the UK [2]. The annual incidence rate is 5.7 per 100,000, representing 1% of all UK cancer incidence [2]. Globally, thyroid cancer incidence rates have increased in recent decades [3], and in the UK are projected to rise by a further 74% between 2014 and 2035 [4]. This has largely been attributed to ‘increased diagnostic scrutiny’ which results in the incidental detection of subclinical disease [5]. However, the incidence of advanced disease and disease-related mortality is also increasing. Lim *et al.* report a 2.9% increase in incidence of advanced-stage papillary thyroid cancer (PTC) and a 1.1% increase in the disease-specific mortality rate between 1994 and 2013 in the USA [3], which is likely to be mirrored in the UK.

Currently in the UK, thyroid cancer is managed by the multidisciplinary team, in accordance with British Thyroid Association guidelines [6]. In the majority of cases, thyroid cancer is managed surgically, with the addition of radioactive iodine ablation (RAI) for more advanced disease. Similarly, the European Society for Medical Oncology advise that surgery is the standard treatment with the addition of RAI for patients with advanced disease or at risk of recurrence [7], while the American Thyroid Association advise that surgery should be the primary treatment modality while RAI, TSH suppression and other treatments can play a role for some patients [8]. Chemotherapy has limited efficacy and is not routinely used [4]. Although differentiated thyroid cancer (DTC; PTC and follicular [FTC] tumors) generally carries a favorable prognosis (98% 5-year survival rate [9]), 20–30% of patients experience disease recurrence [10] and up to 15% of patients will develop treatment refractory disease [11,12]. Treatment refractory disease carries a much poorer prognosis, with 10-year survival rates of only 10% [13,14]. As such, increasing attention is being paid to alternative secondary treatment options for these cancers.

Recently, there has been interest in treatments which specifically target pathways that cause and/or promote DTC. A number of genetic mutations that are associated with DTC development have been identified, including *RET/PTC* translocations, *BRAF*(V600E) point mutations and *RAS* point mutations in PTC [15,16], with *RAS* point mutations and *PPAR-γ* and *PAX8* rearrangements commonly identified in FTC [17]. Many of the identified mutations result in overexpression of activated tyrosine kinases. Following the discovery that tyrosine kinase overexpression is associated with several cancers, tyrosine kinase inhibitors (TKIs) were developed. In addition, overactivation of the VEGF signaling pathways, PI3K/AKT and MAPK, has been demonstrated to promote pathological angiogenesis in both PTC and FTC.

Although the development of TKIs has increased treatment options for recurrent/refractory disease, they have a limited duration of efficacy, meaning that disease progression is delayed, but ultimately not stopped [18]. TKIs are also associated with a significant side effect profile, necessitating discontinuation in a significant number of patients [19]. In the two Phase III clinical trials that studied TKI use in DTC, dose interruptions, reductions or discontinuations were observed in 66.2% of patients treated with sorafenib [20], while discontinuations were observed in 14.2% of patients treated with levantinib [21]. Alternative treatment options are therefore needed, especially with the increasing prevalence of this malignancy.

The current review summarizes the emerging secondary therapeutic options for advanced/treatment refractory DTC. Although a small proportion of DTC progresses to anaplastic carcinoma, its treatment is considered beyond the scope of this review. Particular attention is paid to the current evidence from clinical trials regarding TKIs, and the emerging evidence from preclinical and clinical studies involving immunotherapy.

## Methods

A systematic review was performed to analyze the emerging secondary treatment options for advanced/treatment refractory DTC. Two separate systematic searches of the literature were conducted, following PRISMA guidelines [22], to identify the studies related to TKI and immunotherapeutic treatment modalities. The search terms used were: ‘thyroid AND immunotherapy,’ and ‘thyroid AND TKI OR tyrosine kinase inhibitor.’ Searches were performed by one reviewer using PubMed, Scopus and Google Scholar. Searches were limited to publications in the English language, within between December 2014 and December 2019. Titles, abstracts and full texts of retrieved studies were examined for inclusion. Scientific websites were also consulted (British Thyroid Association [23], American Thyroid Association [8], the US FDA [24], clinical trials website [25]). The reference lists of the selected studies were searched to identify any further relevant papers.

Eligibility criteria included patients/tissues/cells with advanced DTC and treatment with one of the emerging modalities identified. Review articles were used to identify any further relevant original studies, but were not themselves included in the review. Letters to the editor and case studies were excluded. Publications related to TKI were numerous and included several clinical trials. As such the inclusion of publications related to TKI was limited to clinical trials only. Publications related to immunotherapy were far fewer and as such, all relevant publications were included. Data extraction was performed using standardized, predefined and data extraction forms.

## Results

**Figure 1.**
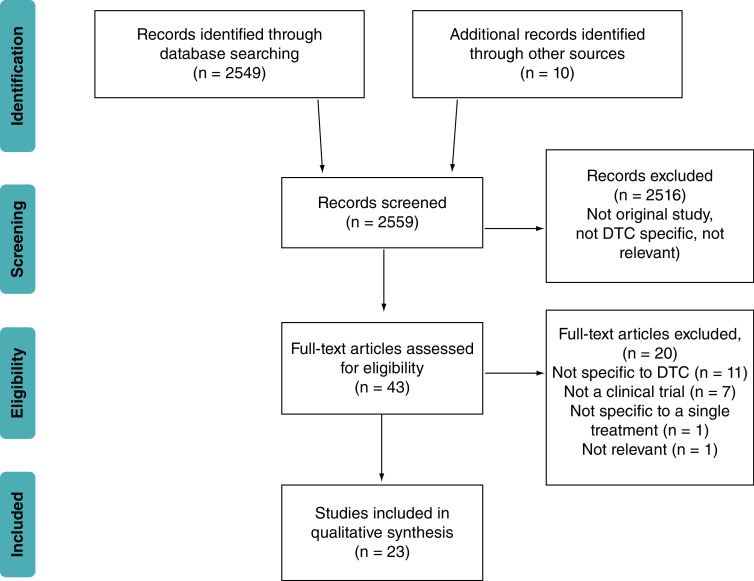
PRISMA diagram showing studies related to tyrosine kinase inhibitors and immunotherapeutic treatment modalities for advanced/treatment refractory differentiated thyroid cancer. The search terms used were: ‘thyroid AND immunotherapy’ and ‘thyroid AND TKI OR tyrosine kinase inhibitor.’ Searches were performed using PubMed, Scopus and Google Scholar. Searches were limited to publications in English between December 2014 and December 2019. DTC: Differentiated thyroid cancer.

The preliminary search identified 2549 publications (Figure 1). A significant proportion of these were not original studies and were excluded. Of these references, 23 matched the inclusion criteria for the review (Table 1). In addition, six ongoing, unpublished clinical trials have been included in the table, which were identified on the clinicaltrials.gov website.

**Table 1.** Studies investigating the effect of tyrosine kinase inhibitors (clinical trials only) and immunotherapeutics (basic research and clinical trials) on thyroid carcinoma.

Study	Design	Participants	Intervention	Outcome	Ref.
Sorafenib in radioactive iodine-refractory, locally advanced or metastatic differentiated thyroid cancer: a randomized, double-blind, Phase III trial (DECISION trial, NCT00984282)	Phase III, multicenter, randomized double-blind clinical trial	Locally advanced or metastatic DTC	Sorafenib (TKI) vs placebo	Longer PFS in the sorafenib group compared with placebo (10.8 vs 5.8 months) More frequent treatment-related adverse event rate in the sorafenib group compared with the placebo group (98.6 vs 87.6%)	[20]
Lenvatinib vs placebo in radioiodine-refractory thyroid cancer (SELECT trial, NCT01321554)	Phase III multicenter, randomized double-blind clinical trial	RAI-refractory progressive DTC	Lenvatinib (TKI) vs placebo	Longer PFS in the lenvatinib group compared with placebo (18.3 vs 3.6 months) 40% treatment-related adverse event rate, 14.2% adverse event-related discontinuation rate and 2% mortality rate in the lenvatinib group	[21]
Vandetanib in locally advanced or metastatic differentiated thyroid cancer: a randomized, double-blind, Phase II trial (NCT00537095)	Phase II, multicenter, randomized, double-blind clinical trial	Locally advanced or metastatic DTC	Vandetanib (TKI) vs placebo	Longer PFS in the vandetanib group compared with placebo (11.1 vs 5.9 months). No statistically significant difference in OS More frequent treatment-related adverse event rate in the vandetanib group. Two treatment-related deaths in the vandetanib group and one in the placebo group	[38]
Vemurafenib in patients with *BRAF* (V600E)-positive metastatic or unresectable papillary thyroid cancer refractory to radioactive iodine: a nonrandomized, multicenter, open-label, Phase II trial (NCT01286753)	Phase II, nonrandomized, multicenter clinical trial	*BRAF*(V600E) positive metastatic or unresectable RAI refractory PTC	Vemurafenib (TKI)	38.5% PR rate, 35% SD rate and 18.2-month median PFS in patients not previously treated with a VEGFR MKI. 27.5% PR rate, 27.5% SD rate and 8.9-month median PFS in patients previously treated with a VEGFR MKI 67% grade 3/4 treatment-related adverse event rate	[51]
A multicenter Phase II study of sunitinib in patients with locally advanced or metastatic differentiated, anaplastic or medullary thyroid carcinomas: mature data from the THYSU study (NCT00510640)	Phase II, multicenter clinical trial	Locally advanced or metastatic thyroid cancer, including 41 participants with DTC	Sunitinib (TKI)	19.5% PR rate, 43.9% SD rate. 13.1-month median PFS and 26.4-month median OS 33% serious treatment-related adverse events rate, and 7% treatment-related deaths	[53]
Efficacy of pazopanib in progressive, radioiodine-refractory, metastatic-differentiated thyroid cancers: results of a Phase II consortium study	Phase II, single-arm study	Progressive, RAI-refractory, metastatic DTC	Pazopanib (TKI)	49% PR rate 47% PFS and 81% OS at 1 year 43% adverse event-related dose reduction	[56]
Phase II study evaluating the combination of sorafenib and temsirolimus in the treatment of radioactive iodine-refractory thyroid cancer	Phase II, single-arm clinical trial	RAI-refractory FTC	Sorafenib (TKI) and temsirolimus (mTOR inhibitor) in combination	22% PR rate, 58% SD rate. 30.5% PFS rate at 1 year 64% grade 3+ treatment-related adverse event rate, 61% adverse event-related dose reduction, one ‘possible’ treatment related death	[59]
Cabozantinib as salvage therapy for patients with tyrosine kinase inhibitor-refractory differentiated thyroid cancer: results of a multicenter Phase II international thyroid oncology group trial (clinicaltrials.gov, 2018b), (NCT01811212)	Phase II, nonrandomized single-arm clinical trial	RAI-refractory DTC with previously failed first-line VEGFR-targeted TKI treatment	Cabozantinib (TKI)	40% PR rate, 52% SD rate. 12.7-month median PFS and 34.7-month median OS	[30]
Role of salvage-targeted therapy in differentiated thyroid cancer patients who failed first-line sorafenib	Retrospective review	RAI-refractory DTC with previous failed sorafenib treatment	Sorafenib (TKI) monotherapy vs sorafenib followed by salvage second-line TKI	100% SD or PR rate with salvage TKI therapy Longer OS in the salvage therapy group compared with the control group (58 vs 28 months)	[62]
Evaluation of efficacy, safety of vandetanib in patients with differentiated thyroid cancer (VERIFY trial, NCT01876784)	Phase III, multicenter-randomized, double-blind, clinical trial	Locally advanced, metastatic or RAI-refractory DTC	Vandetanib (TKI) vs placebo	Longer PFS in the vandetanib group compared with placebo (10.0 vs 5.7 months Formal publication pending	[40]
A Phase II trial of cabozantinib for the treatment of radioiodine (RAI)-refractory differentiated thyroid carcinoma (DTC) in the first-line setting (NCT02041260)	Phase II clinical trial	Locally advanced, metastatic or RAI-refractory thyroid cancer, including 22 participants with DTC	Cabozantinib (TKI)	PR rate of 54% and SD rate of 43%. PFS and OS not reported No significant treatment-related adverse events or discontinuations Outcomes from preliminary results, formal publication pending	[41]
Study of apatinib in patients with differentiated thyroid cancer (clinicalTrials.gov), (NCT02731352)*	Phase II clinical trial	DTC	Apatinib (TKI)	Pending	
Phase II trial of apatinib mesylate in locally advanced/metastatic differentiated thyroid carcinoma (NCT0316738)*	Phase II clinical trial	Advanced or metastatic DTC	Apatinib (TKI)	Pending	[47]
Study of neoadjuvant regimen for radioactive iodine treatment of metastatic and advanced differentiated thyroid cancers (APT-01), (NCT04180007)*	Phase II clinical trial	Advanced or metastatic DTC	Apatinib (TKI)	Pending	[49]
Efficacy of apatinib in radioactive iodine-refractory differentiated thyroid cancer (NCT03048877)*	Phase III clinical trial	RAI-refractory DTC	Apatinib (TKI)	Pending	[50]
Safety and antitumor activity of the anti-PD-1 antibody pembrolizumab in patients with advanced, PD-L1-positive papillary or follicular thyroid (KEYNOTE-28 trial, NCT02054806)	Phase Ib, single-arm clinical trial	Advanced, PD-L1-positive DTC that had not responded to standard therapy	Pembrolizumab (anti-PD-1 antibody)	9.1% PR rate, 54.5% SD rate. 7-month median PFS One ≥grade 3 treatment-related adverse event rate	[81]
Combining *BRAF* inhibitor and anti-PD-L1 antibody dramatically improves tumor regression and antitumor immunity in an immunocompetent murine model of anaplastic thyroid cancer	*In vitro* study – murine model	*BRAF*-V600E-mutated thyroid cell lines	Anti-PD-L1 antibody monotherapy vs anti-PD-L1 antibody therapy in combination PLX4720 (a *BRAF* inhibitor)	No tumor volume reduction with anti-PD-L1 antibody monotherapy. Greater tumor volume reduction with combined anti-PD-L1 antibody and PLX4720 therapy compared with PLX4720 monotherapy	[79]
Study of pembrolizumab (MK-3475) in participants with advanced solid tumors (MK-3475-158/KEYNOTE-158), (NCT02628067)	Nonrandomized, parallel assignment	Solid tumors including a cohort with thyroid cancer	Pembrolizumab (anti-PD-1 antibody)	Pending	[82]
Durvalumab plus tremelimumab for the treatment of patients with progressive, refractory advanced thyroid carcinoma – the DUTHY trial (DUTHY), (NCT03753919)	Phase II clinical trial	Progressive, advanced thyroid cancer, Including one cohort with DTC	Durvalumab (PD-L1 inhibitor) in combination with tremelimumab (CTLA-4 inhibitor)	Pending	[74]
Phase I dose-escalation study of VB-111, an antiangiogenic virotherapy, in patients with advanced solid tumors	Phase I clinical trial	Advanced solid tumors, including one participant with PTC	VB-111 (an engineered antiangiogenic adenovirus)	>30% reduction in the diameter of the thyroid mass and some central necrosis. SD for 18 months No significant treatment-related adverse events	[99]
Antitumor activity of VB-111, a novel antiangiogenic virotherapeutic, in thyroid cancer xenograft mouse model	*In vitro* study – xenograft nude mouse model	Tumor nodules grown from thyroid cancer cell lines, including two DTC cell lines	VB-111 (an engineered antiangiogenic adenovirus)	26.6% rate of tumor growth inhibition in FTC tumors. 34.4% rate of tumor growth inhibition in PTC tumors No nonspecific toxicity	[97]
Treatment of aggressive thyroid cancer with an oncolytic herpes virus	*In vitro* and *in vivo* study	Thyroid cancer cell lines, including two DTC cell lines	NV1023 (an attenuated, replication competent HSV)	Almost complete death of PTC cell line by day 7 at 0.1 MOI No significant cytotoxicity in FTC cell line at MOI 0.1 or 1. 40.6% viability by day 7 at MOI 5 100% rate of complete regression of PTC cell line tumors in nude mice, following NV1023 injection	[95]
Treatment of human thyroid carcinoma cells with the g47delta oncolytic herpes simplex virus	*In vitro* study	Thyroid cancer cell lines, including two DTC cell lines	G47Δ (a third-generation HSV vector)	>30% and >80% of PTC cells were killed by day 5 at 0.01 MOI and 0.1 MOI, respectively >21% and >38% of FTC cells were killed by day 5 at 0.01 MOI and 0.1 MOI, respectively	[98]
Preoperative recombinant adenoviral human *p53* gene combined with intensity-modulated radiation therapy in treatment of stage IV papillary thyroid carcinoma: a randomized clinical study	Randomized clinical trial	Advanced (stage IV) PTC	rAd-P53 (recombinant adenoviral human *p53*) gene therapy in combination with radiation therapy vs radiation monotherapy	Greater resectability rate in the gene therapy in combination with radiation therapy vs radiation therapy (76 vs 47.6%) Greater PR rate in patients with nonresectable disease in the gene therapy in combination with radiation therapy vs radiation therapy (100 vs 72%) Results from poster presentation at the 2012 American Society of Oncology meeting Formal publication pending	[101]
Safety and efficacy of VB-111 in subjects with advanced differentiated thyroid cancer (NCT01229865). Results not yet published	Phase II, nonrandomized single-group assignment	Advanced DTC	VB-111 (an engineered antiangiogenic adenovirus)	Pending	[100]
*rAd-p53* gene therapy for advanced malignant thyroid tumors (NCT00902122)	Phase IV, multicenter, randomized clinical trial	Advanced thyroid cancer	*rAd-p53* (recombinant adenoviral human *p53*) monotherapy vs rAd-p53 with RAI vs rAd-p53 with RAI and surgery	Pending	[102]
Redifferentiation of iodine-refractory *BRAF*-V600E-mutant metastatic papillary thyroid cancer with dabrafenib	Phase II clinical trial	*BRAF*-V600E-mutated RAI-refractory PTC	Dabrafenib (selective *BRAF* inhibitor)	60% rate of RAI resensitization allowing further RAI treatment. 67% SD rate and 33% PR rate following further RAI treatment 80% grade 1 + 2 treatment-related adverse effects. No serious treatment-related adverse events, dose reductions or discontinuations	[106]
Vemurafenib redifferentiation of *BRAF* mutant, RAI-refractory thyroid cancers	Single-center pilot trial	RAI-refractory *BRAF*-V600E-mutated FTC	Vemurafenib (*BRAF* inhibitor)	40% rate of sufficiently increased RAI avidity to allow further RAI treatment. 100% of patients receiving further RAI showed PFS at 6 months 90% grade 1–3 treatment-related adverse event rate. No serious treatment-related adverse events, discontinuations or fatalities	[31]
CAR-T therapy targeting ICAM-1 eliminates advanced human thyroid tumors	*In vitro* study	Advanced thyroid cancer cell lines, including PTC	T cells transduced with the anti-ICAM-1 CAR	‘Robust and specific killing’ of PTC cell lines	[87]

Studies have been grouped according to treatment modality. For each modality, clinical studies are listed primarily (Phase III, followed by Phase II), followed by in vitro studies, in vivo studies and finally ongoing or unpublished studies. Where there is more than one of each study type, they are listed with the most recent first.

*Ongoing trial.

CAR-T: Chimeric antigen receptor T cell; CTLA-4: Cytotoxic T lymphocyte antigen 4; DTC: Differentiated thyroid cancer; FTC: Follicular thyroid cancer; HSV: Herpes simplex virus; ICAM-1: Intercellular adhesion molecule 1; MKI: Multikinase inhibitor; MOI: Multiplicities of infection; OS: Overall survival; PD-1: Programmed cell death protein-1; PD-L1: programmed cell death ligand-1; PFS: Progression-free survival; PR: Partial response; PTC: Papillary thyroid cancer; RAI: Radioactive iodine ablation; SD: Stable disease; TKI: Tyrosine kinase inhibitor.

## Tyrosine kinase inhibitors

**Figure 2.**
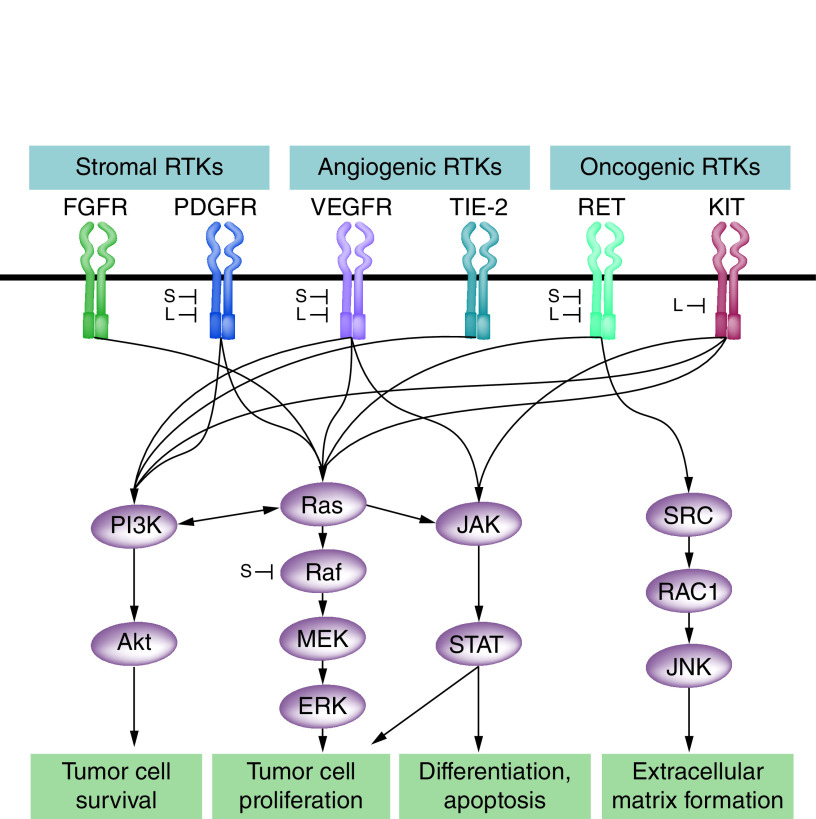
Lenvatinib and sorafenib inhibit multiple pathways involved in differentiated thyroid cancer development and progression. L: Lenvatinib; RTK: Receptor tyrosine kinase; S: Sorafenib. Adapted with permission from [109].

The treatment of several cancers has been revolutionized by the advent of targeted therapy with TKIs, which selectively target the signaling pathways that promote carcinogenesis (Figure 2). Multikinase inhibitors (MKI) simultaneously target a number of different pathways, meaning that they effectively inhibit several different pathways in parallel. The FDA has approved the use of sorafenib and lenvatinib for the treatment of advanced DTC [26]. A number of oncogenic drivers in DTC have been described, including RET/PTC, RAS, *BRAF* and NTRK1 [27]. The advances in genomic profiling open the possibility of treating patients with TKIs that specifically target the responsible mutation implicated in a patient’s cancer, but work is at an early stage. In 2018, the FDA [24] approved the use of the NTRK1-3 inhibitor larotrectinib for the treatment of patients with solid tumors that have an *NTRK* gene fusion, following positive results from three multicenter, open-label, single-arm trials [28]. Although data from large trials investigating TKI efficacy in the treatment of DTC harboring specific mutations are lacking, there are favorable outcomes reported in small studies. Ho *et al.* [29] initially reported that selumetinib increased RAI uptake in five of five participants with NRAS-positive RAI-resistant DTC, compared with four of nine patients with *BRAF* mutations, suggesting efficacy may be greater in patients with RAS mutant DTC; Cabanillas *et al.* [30] reported an ORR of 90% in 21 thyroid cancer patients (pooled data from two trials); and most recently Dunn *et al.* [31] reported that vemurafenib restored RAI uptake and efficacy in four of ten participants with RAI-resistant thyroid cancer. Interestingly, an analysis of the gene expression profiles from the tumor samples of participants involved in the Phase III DECISION trial [20,32] did not identify variations in sorafenib efficacy across expression profile groups [33].

### FDA approved TKI for clinical use in DTC

#### Sorafenib

Sorafenib is an MKI that targets *BRAF*, VEGFR, PDGFR-β and RET. Brose *et al.* [20,32] conducted a multicenter randomized double-blind Phase III trial (DECISION, NCT00984282) assessing the efficacy and safety of orally administered sorafenib in RAI refractory locally advanced or metastatic DTC. They randomized 417 patients to receive sorafenib (n = 207) or placebo (n = 210) and demonstrated a significantly longer progression-free survival (PFS) in the sorafenib group (10.8 months) compared with the placebo group (5.8 months). Although there was no change in overall survival (OS), this was partly attributed to the study design that allowed for crossover of patients from the placebo group into the treatment group on progression of disease. Treatment-related adverse events (most commonly grade 1 or 2, including hand–foot skin reaction, diarrhea, alopecia and rash) occurred in 98.6% of patients receiving sorafenib compared with 87.6% of people receiving placebo.

#### Lenvatinib

Lenvatinib is an MKI that targets VEGFR, PDGFRα, RET and KIT. Schlumberger *et al.* [21,34] conducted a multicenter, randomized, double-blind, Phase III trial (SELECT, NCT01321554) assessing the efficacy and safety of this agent in patients with RAI-refractory progressive DTC. Schlumberger *et al.* randomized 392 patients to receive lenvatinib (n = 261) or placebo (n = 131). A significantly longer PFS was demonstrated in the lenvatinib group (18.3 months) than in the placebo group (3.6 months). At the time of primary data cutoff, median OS had not been reached. Treatment-related adverse events occurred in more than 40% of treated patients (most commonly, diarrhea, hypertension, proteinuria and decreased appetite). This resulted in an adverse event-related discontinuation rate of 14.2% for treated patients, and a mortality rate of 2%.

In a subsequent data analysis, Brose *et al.* stratified participants by age (<65 or >65 years) and demonstrated a significantly longer OS in participants more than 65 years treated old with lenvatinib compared with those in the placebo arm (hazard ratio: 0.53; 95% CI: 0.31–0.91; p = 0.020) [35]. Furthermore, although participants less than 65 years showed longer OS in the placebo arm (p = 0.018), there was no statistically significant difference in OS between age groups in the lenvatinib arm (p = 0.082).

The National Comprehensive Cancer Network recommends either sorafenib or lenvatinib for the treatment of progressive or advanced RAI refractory DTC [36]. There is currently insufficient evidence to conclude which drug offers a better treatment option as the two Phase III trials considering the efficacy and safety of each, described above, are not directly comparable owing to significant differences in the study design and participants involved [37]. Consequently, there is no guidance as to which drug to use and when.

### TKI investigated for clinical use in DTC but not currently FDA approved

#### Vandetanib

Vandetanib is an MKI that targets VEGFR, EGFR and RET-tyrosine kinase. Leboulleux *et al.* [38,39] conducted a multicenter, randomized, double-blind Phase II trial (NCT00537095) assessing the efficacy of vandetanib in patients with metastatic or locally advanced DTC. Leboulleux *et al.* randomized 145 patients to receive vandetanib (n = 72) or the placebo (n = 73). They demonstrated a significantly longer PFS in the vandetanib group (11.1 months) than in the placebo group (5.9 months). Increased PFS was most significant in the subgroup of participants with PTC (16.2 months). There was no statistically significant difference demonstrated in OS between the vandetanib group and the placebo group. Treatment-related adverse events frequently observed in the treatment group (QTc prolongation, an abnormality in cardiac conduction 14%, diarrhea 10%, asthenia 7% and fatigue 5%) and were uncommon in the placebo group (asthenia 4%). There was one treatment-related death in the placebo group and two in the vandetanib group.

A multicenter randomized double-blind, Phase III trial (VERIFY, NCT01876784) assessing the efficacy of vandetanib in patients with locally advanced, metastatic or RAI refractory DTC was completed in 2015 [40]. This demonstrated longer PFS in the vandetanib group (10.0 months) compared with the placebo group (5.7 months). OS data are not yet available, and the study is yet to be published.

#### Cabozantinib

Cabozantinib is an MKI that targets VEGF receptor kinase, RET, MET and AXL. Brose *et al.* [41] conducted a Phase II, single-arm trial (NCT02041260) assessing the efficacy of cabozantinib in patients with metastatic, RAI-refractory, unresectable or locally advanced thyroid cancer. The primary study completion date was March 2020, and although the final results are yet to be formally published, interim results were presented by poster at the 2018 American Society of Clinical Oncology annual meeting [42]. Thirty-five patients, including 22 with DTC were recruited with a partial response (PR) being demonstrated in 54% and stable disease (SD) in 43% of patients. PFS and OS results were not reported and there were no significant treatment-related adverse events or discontinuations.

A Phase III multicenter randomized, double-blind, placebo-controlled trial (COSMIC-311, NCT03690388) assessing the efficacy of cabozantinib on PFS and objective response rate in participants with RAI refractory DTC who have progressed after prior VEGFR-targeted therapy is currently ongoing in the USA [43]. Formal publication of results is pending, however, in late 2020 the biotechnology company Exelixis (CA, USA) announced results from an early interim analysis that demonstrated ‘significant improvement’ in PFS, with cabozantinib reducing the risk of disease progression or death by 78%. Consequently, it was recommended that further enrollment be stopped and sites and patients be unblinded [44].

#### Apatinib

Apatinib is a TKI that targets VEGFR-2. Lin *et al.* [45] conducted a small, single-arm, prospective study that evaluated the metabolic and structural responses to apatinib in ten patients with RAI refractory PTC. All had undergone total thyroidectomy and had evidence of pulmonary metastases. Lin *et al.* evaluated the total tumor diameter (defined as the sum of the diameter of target lesions) of 18 pulmonary metastatic lesions and found that there was a statistically significant reduction 8 weeks following treatment (44.7–22.8 mm; p = 0.001). According to the RECIST 1.1 criteria [46], nine patients were shown to have achieved a PR and one SD. No severe treatment-related adverse events were observed.

Presently, there are three active Phase II clinical trials (NCT02731352 [47], NCT03167385 [48], NCT04180007 [49]) and one Phase III (NCT03048877 [50]) clinical trial that consider the efficacy of apatinib in DTC. At the time of writing, no results had been posted.

#### Vemurafenib

Vemurafenib is a TKI that targets mutated *BRAF*-V600E kinase. Brose *et al.* [51] conducted a nonrandomized, multicenter, open-label Phase II trial (NCT01286753 [52]) assessing the efficacy and safety of Vemurafenib in 51 patients with *BRAF* (V600E)-positive metastatic or unresectable RAI refractory PTC. Participants were studied in two distinct cohorts, according to whether they had been previously treated with a VEGFR MKI (cohort 2, 25 patients) or not (cohort 1, 26 patients). Seventy-three percent of patients in cohort 1 achieved disease control, 38.5% of whom showed a PR and 35% achieved SD. Median PFS was 18.2 months but OS had not been reached at the time of publication, and no further updates have been issued. In contrast, 55% of patients in cohort 2 achieved disease control; half of whom showed PR and half achieved SD. Median PFS was 8.9 months and Grade 3 or 4 treatment-related adverse events were recorded in 67% of patients across both cohorts.

#### Sunitinib

Sunitinib is an MKI that targets PDGFR, VEGFR, c-KIT and RET. Ravaud *et al.* [53] conducted a single-arm, multicenter, open-label Phase II trial (THYSU, NCT00510640 [54]) assessing the efficacy and safety of sunitinib in 71 patients with locally advanced or metastatic thyroid cancer, including 41 patients with DTC. One patient (2.4%) achieved a complete response, 19.5% showed a PR and 43.9% achieved SD. Median PFS was 13.1 months and median OS was 26.4 months. Thirty-three percent of all participants experienced serious treatment-related adverse events, and there were five (7%) treatment-related deaths.

Currently, there is an ongoing Phase II, single-arm trial (NCT00381641 [55]) investigating the safety and efficacy of sunitinib in advanced thyroid cancer, including a cohort of 38 patients with RAI refractory DTC. All study participants received sunitinib. Patients in the DTC cohort demonstrated an objective response rate of 23.7% and an OS of 32.6 months. There was a 44.74% incidence of serious adverse events. The results are yet to be formally published.

#### Pazopanib

Pazopanib is a TKI that targets VEGRE, PDGFR and c-KIT. Bible *et al.* [56] conducted a Phase II, single-arm study considering its efficacy in 39 patients with progressive, RAI refractory, metastatic DTC in 2010. Although there were no complete responses, a PR was seen in 18 patients (49%). Of the 17 patients (46%) who continued with pazopanib for 1 year or longer, median OS and PFS at 1 year were 81 and 47%, respectively. Adverse event-related dose reductions were seen in 16 (43%) patients (most frequently fatigue, hypopigmentation of hair and skin, diarrhea and nausea). Deaths were seen in two patients who had a pre-existing contributory disorder. Despite the promising results demonstrated the study was not taken to the Phase III stage, however, the reason for this is not clear.

More recently, a Phase II clinical trial (NCT00625846 [57]) considering the safety and efficacy of pazopanib in advanced thyroid cancer, including a cohort of 39 patients with DTC was completed in February 2020. All study participants received pazopanib. Patients in the DTC cohort demonstrated an overall response rate (ORR) of 49% and a PFS of 71 %. There was a 40% incidence of grade 3+ treatment-related adverse events. The results are yet to be formally published.

#### Larotrectinib

Larotrectinib is a selective tropomyosin receptor kinase inhibitor. Cabanillas *et al.* analyzed the effectiveness of larotrectinib in treating participants with *NTRK* positive locally advanced or metastatic thyroid cancer from the pooled data of two clinical trials involving participants with solid tumors. Although the results are yet to be formally published, they were presented by poster at the 2020 European Society for Medical Oncology virtual congress [58]. They analyzed the responses of 28 participants with thyroid cancer, including 21 with DTC. An ORR of 90% was observed in participants with DTC; 10% showed complete response, 81% PR and 10% SD. Grade ≥3 treatment-related adverse events were seen in 7% of participants.

### Combination TKI therapy

Sherman *et al.* [59] conducted a single-center, single-arm, Phase II trial assessing the efficacy of the combination of sorafenib and temsirolimus in the treatment of RAI refractory FTC. Temsirolimus inhibits mammalian target of rapamycin (mTOR), a kinase involved in cell proliferation and autophagy. Disrupted mTOR signaling reduces the proliferative effect of thyroid-stimulating hormone and impairs thyroid cancer growth [60]. Sherman *et al.* treated 36 patients with oral sorafenib and intravenous temsirolimus. Fifty-six percent of study participants had received prior systemic or targeted therapy (including sorafenib). An overall PR rate of 22% was observed, and SD was seen in 58% of patients. PR rates were greater in patients who had not received prior systemic treatment (38 vs 10%). The PFS at 1 year was 30.5%. Significant (≥grade 3) treatment-related adverse events were seen in 64% of patients (most commonly hyperglycemia, fatigue, anemia and oral mucositis) necessitating a dose reduction in one or both drugs in 61% of participants. There was one ‘possible’ treatment-related death.

### Salvage TKI therapy

Unfortunately, TKIs have a limited duration of efficacy as described previously and are associated with significant treatment-related adverse events, often resulting in discontinuation [19]. Currently, there are no approved second- or third-line options following discontinuation of first-line regulatory approved therapy, and such treatments are only provided off license or as part of a clinical trial [61].

Dadu *et al.* [62] conducted a retrospective review studying the efficacy of alternative, second-line TKI in patients with RAI refractory DTC who experienced treatment failure with sorafenib. Participants in the control group (n = 35) received sorafenib alone, while those in the treatment group (n = 25) received sorafenib followed by salvage therapy with either sunitinib, pazopanib, cabozantinib, lenvatinib or vemurafenib. All drugs used in the treatment group have a similar mechanism of action to sorafenib. All patients who had disease progression with sorafenib demonstrated a PR or SD with salvage TKI therapy. Similarly, OS was significantly longer in the salvage therapy group (58 months) compared with the control group (28 months). Interestingly, two patients who had treatment-related discontinuations with sorafenib tolerated and responded to salvage sunitinib.

Subsequently, Cabanillas *et al.* [30] conducted a nonrandomized, single-arm, Phase II trial (NCT01811212 [41]) investigating the efficacy of cabozantinib in RAI refractory DTC that had progressed on any first-line VEGFR-targeted TKI treatment. Of the 25 enrolled participants, 84% had received one prior VEGFR-targeted therapy, while 16% had received two therapies. Cabozantinib targets c-MET, RET and VEGFR. Importantly, c-MET is implicated in VEGFR inhibitor resistance [30]. Forty percent of patients demonstrated a PR, 52% had SD and 8% had disease, which could not be evaluated. PFS was 12.7 months and OS was 34.7 months. One treatment-related death was observed.

For patients who progress on, or who are unable to tolerate first-line TKI, salvage TKI therapy may offer a promising solution where treatment options are otherwise limited. Larger, Phase III trials are needed.

## Immunotherapy

It is well recognized that the immune system mounts an anticancer response to neoplastic cells [63]. This is achieved by the production of immunity to distinctive antigenic peptides present on cancer cells [1]. However, tumors can trigger a number of mechanisms to avoid recognition and elimination by the immune system through a process known as ‘immune escape’. In general, immunotherapy works by over-riding the pathways that lead to immune escape, thereby reactivating the antitumor immune response.

In recent years, there has been progress in the development of immunotherapy for the treatment of various cancers, in particular hematological malignancies [64,65]. However, the complex microenvironment of solid tumors and the off target toxicity that results from the presence of solid tumor antigens in somatic tissues has made the development of immunotherapy for solid cancers more challenging [66]. Published research regarding immunotherapy for the treatment of thyroid cancer specifically is limited and is largely preclinical [1].

### Immune checkpoint inhibitors

Immune checkpoints are inhibitory pathways that control the severity of an immune response in order to limit damage to healthy cells. One method of tumor immune escape is by hijacking these checkpoint pathways and downregulating the specific, antitumor immune response [67,68]. Partner proteins/ligands on the surface of, or secreted by, neoplastic cells bind with immune checkpoint receptors on T cells, effectively acting as an ‘off switch’, which prevents the T cells from destroying the neoplastic cells. Checkpoint inhibitors block the ‘off switch’ allowing T cells to remain effective against neoplastic cells [69]. There are two major classes of checkpoints that regulate different stages of the immune response; those which target cytotoxic T lymphocyte antigen 4 (CTLA-4) and those which target programmed cell death protein-1 (PD-1) or its ligand (PD-L1) [70]. Although immune checkpoint inhibitors have revolutionized the treatment of many malignancies, there is varying success depending on tumor type and the drug used and many trials are ongoing [71].

#### Checkpoint inhibitors against CTLA-4

CTLA-4 principally modulates the immune response against infective agents in draining lymph nodes. T-cell exposure to antigens by antigen-presenting cells in lymph nodes results in T-cell activation and overexpression of surface CTLA-4. CTLA-4 competes with the T-cell activation molecule CD28 for binding to CD80 or CD86 on the surface of antigen-presenting cells promoting immune suppression [72]. While CTLA-4 inhibitors have proved revolutionary in the treatment of some cancers, for example, melanoma [73] research regarding their use in DTC is in its infancy. A literature search did not identify any preclinical studies involving CTLA-4 inhibitors on thyroid cancer tissue or cells. At the time of writing, there were 60 ongoing clinical trials considering CTLA-4 inhibitors in solid tumors, two of which specifically listed thyroid cancers. The only clinical trial exclusively considering thyroid cancer is investigating a combination of a PD-L1 and a CTLA-4 inhibitor. This prospective, Phase II trial (DUTHY, NCT03753919 [74]) aims to evaluate the efficacy and safety of Durvalumab (PD-L1 inhibitor) with tremelimumab (CTLA-4 inhibitor) in three different patient cohorts with DTC, ATC and MTC; study completion is expected in 2021.

#### Checkpoint inhibitors against PD-L1

Activation of T cells results in the surface expression of PD-1 receptors. Dendritic cells and macrophages express ligands for PD-1, PD-L1 and PD-L2. The PD-1/PD-L1 pathway is inhibitory; its activation regulates the T-cell response that prevents uninhibited inflammation and autoimmunity [69]. PD-L1 is frequently overexpressed by neoplastic cells and has been demonstrated to aid immune escape in a number of different cancers [71,75]. T-cell PD-1 receptors bind to PDL-1 on tumor cells, resulting in inhibition of the cytotoxic response and promotion of immune escape.

Cunha *et al.* have demonstrated that thyroid cancer cells demonstrate higher levels of PD-L1 compared with benign tumors [76]. In addition, more advanced PTC expressed elevated PD-L1 mRNA compared with less advanced tumors, suggesting PD-L1 may represent a useful target in thyroid cancer checkpoint inhibition.

The most recognized gene mutation in the development of PTC is the* BRAF(*V600E) mutation [77]. Angell *et al.* [78] demonstrated that PTC expressing the *BRAF*(V600E) mutation frequently express PD-L1. Subsequently, Brauner *et al.* [79] demonstrated that *BRAF*(V600E)*-*mutated thyroid cell lines and tumor specimens demonstrated higher baseline PD-L1 mRNA expression than *BRAF*-*WT* thyroid cells and tumor specimens in a murine thyroid cancer model. They showed that SCID mice injected with human thyroid cancer cells showed no tumor volume reduction with anti-PD-L1 treatment alone, but combination treatment using the *BRAF*(V600E) inhibitor, PLX4720 [80] and anti-PD-L1 antibody worked synergistically to cause greater tumor volume reduction compared with treatment with PLX4720 alone. Furthermore, Severson *et al.* [80] studied the immune response *in vitro* in cells harvested from lymph node metastases of patients with DTC and found that PD-1^+^CD4^+^ cells and PD-1^+^CD8^+^ cells were enriched in 67%. However, the enriched PD-1^+^CD8^+^ cells demonstrated a reduced cytotoxic function and reduced ability to produce IL-2 and TNF-α when compared with enriched lymphocyte cells obtained from control lymph nodes. This suggests that lymph nodes provide an inflammatory microenvironment that promotes an antitumor immune response, but this is limited in effectiveness. As such they suggested that immunotherapies that inhibit PD-1/PD-L1 may represent an effective future treatment option for patients with DTC and lymph node metastasis.

Pembrolizumab is an anti-PD-1 antibody that promotes antitumor activity by blocking PD-1 and therefore preventing ligand binding. Its efficacy in the treatment of several different cancers has been demonstrated and it is currently approved by the FDA for use in the treatment of ten different cancers including head and neck squamous cell carcinoma and melanoma [81]. Mehnert *et al.* conducted a single-arm, Phase Ib trial (KEYNOTE-28, NCT02054806 [82]) to evaluate the safety and antitumor activity of pembrolizumab in 22 patients with advanced DTC that demonstrated PD-L1 expression in tumor or stromal cells, and had not responded to standard therapy. Eighteen patients experienced mild treatment-related adverse events, and one patient experienced a grade ≥3 treatment-related adverse event. However, no treatment-related discontinuations or deaths were observed. Two patients (9.1%) demonstrated a PR for 8 and 20 months. Just over half (54.5%) of patients demonstrated SD and the median PFS was 7 months. These results were sufficiently positive for inclusion of DTC in the on-going multicohort Phase II trial (KEYNOTE-158, NCT02628067 [83]) of pembrolizumab, which is scheduled to finish in 2026.

### Chimeric antigen receptor T cell therapy

Chimeric antigen receptor T (CAR-T) cells are genetically engineered T cells expressing T-cell receptor constructs that not only target specific tumor-associated antigens but possess intracellular signaling motifs [84]. Although CAR-T-cell therapy has shown success in the treatment of hematological malignancies [85], there has been less success demonstrated in treating solid tumors [86], and there are no clinical studies considering thyroid carcinoma specifically.

*In vitro*, Min *et al.* [87] have demonstrated that CAR-T cells kill advanced thyroid carcinoma cell lines (8505C, BCPAP, FRO and KHM-5M). The authors report that PTC is associated with increased expression of intercellular adhesion molecule 1 (ICAM-1). T cells were transduced with the anti-ICAM-1 CAR and their cytotoxicity against the ICAM-1^+^ thyroid cancer cell lines was evaluated. A ‘robust and specific killing’ was demonstrated when using CAR-T cells that should encourage further research into CAR-T cell therapies for advanced thyroid cancer [88].

### Vaccines

Therapeutic vaccines aim to treat cancers through encouraging the immune system to destroy neoplastic cells and preserve normal cells. This can be achieved with autologous tumor vaccines using whole tumor cells and dendritic cells, and with recombinant vaccines based on peptides from defined tumor-associated antigens [89].

Cancer/testis antigens are types of antigenic peptides that have been increasingly utilized as potential targets for vaccine-based immunotherapy for different cancer types over the past few years [66]. NY-ESO-1 is a cancer/testis antigen expressed in several different cancer types including sarcoma, melanoma and ovarian cancers [66] that have been shown to be a promising target for immunotherapy in clinical studies [90]; It has been used in vaccines where NY-ESO-1-derived peptides are used to pulse dendritic cells that are then added to the tumor to directly stimulate the T cells present. Research regarding NY-ESO-1 in thyroid cancer is only at the preclinical stage. Gunda *et al.* [1] analyzed the baseline expression of NY-ESO-1 in multiple thyroid cancer cell lines. Although this was found to be low across all tumors cell lines, the use of the demethylating agent deoxyazacytidine (DAC) significantly boosted expression throughout. In addition, they found that DAC-induced NY-ESO-1 gene expression was reduced by the use of the *BRAF* inhibitor, PLX4720 [79] in some *BRAF* mutant thyroid cancer cell lines. Similarly, raised NY-ESO-1 expression was demonstrated in the orthotopic thyroid tumors of SCID mice given short-term DAC treatment. When untreated or DAC-treated thyroid cancer cell lines were cocultured with peripheral blood lymphocytes transduced with NY-ESO-1 T-cell receptors, the T cells in the treated group showed significant increases in IFN-γ and Granzyme-B; signifying T-cell recognition of tumor cells, which may represent a potential new treatment pathway.

### Oncolytic virotherapy

Oncolytic viruses are viruses that selectively infect and destroy cancer cells, without damaging normal cells [91]. Oncolytic viruses can be naturally oncolytic, termed ‘wild type’ or can be genetically modified to become oncolytic through genome manipulation [92]. Oncolytic virotherapy has proved efficacious in the treatment of a number of cancers. In particular, talimogene laherparepvec, a genetically modified herpes simplex virus (HSV), has demonstrated overwhelming success in the treatment of melanoma and is now approved for its treatment by the FDA [93,94]. Research regarding oncolytic virotherapy in DTC has shown less progress; only one completed preclinical study within the search dates was identified, and only a handful published prior to this [95–98].

#### Herpes simplex virus

Yu *et al.* [95] investigated the activity of NV1023, an attenuated, replication-competent HSV against seven different thyroid cancer cell lines, including one PTC (NPA-187) and one FTC (WRO82-1) cell line. NV1023 was added to plated cell lines at multiplicities of infection (MOI) of 0, 0.1, 1 and 5, and cell survival was assessed every 2 days. The PTC cell line was sensitive to NV1023 cytotoxicity, demonstrating almost complete cell death by day 7 at 0.1 MOI. The FTC cell line was comparatively resistant, with no significant cytotoxicity demonstrated at MOI 0.1 or 1. A modest, late effect was demonstrated at MOI 5, with 40.6% viability at day 7.

The therapeutic efficacy of NV1023 was further assessed *in vivo* in several of the thyroid cancer cell lines, including the PTC cell line; thyroid cancer tumors were established in the flanks of nude mice and injected with NV1023. All PTC cell line tumors (6/6) regressed completely after a single injection of NV1023, in contrast to controls.

More recently, Wang *et al.* [98] investigated the therapeutic effects of a third-generation HSV vector, G47Δ, on different thyroid cancer cell lines, including a PTC (KAT-5) and an FTC (WRO). Cancer cells were cultured and seeded and then infected with G47Δ at MOI of 0.01 and 0.1; their survival was monitored daily. By day 5, greater than 30 and 80% of PTC cells were killed at 0.01 MOI and 0.1 MOI, respectively. FTC cells displayed less sensitivity to G47Δ; at the same point, 21 and 38% of cells were killed at 0.01 and 0.1 MOI, respectively.

#### Adenovirus

In 2011, Reddi *et al.* [97] evaluated the efficacy of the engineered antiangiogenic adenovirus VB-111 in thyroid cancer by using a xenograft nude mouse model with several thyroid cancer-derived cell lines, including both FTC and PTC. Tumor nodules were grown in the animals followed by intravenous injection of VB-111 suspension. This resulted in a 26.6% (p = 0.060) and 34.4% (p = 0.005) rate of tumor growth inhibition in FTC and PTC tumors, respectively, with no observed nonspecific toxicity.

In 2013, Brenner *et al.* [99] conducted a Phase I study considering the effectiveness of VB-111 in 33 patients with advanced solid tumors, including one patient with PTC. The patient with PTC responded well; CT scanning at 6 and 12 months following treatment with VB-111 demonstrated a more than 30% reduction in the diameter of the thyroid mass and some central necrosis and the patient had SD for 18 months. Subsequently, they showed disease progression and were treated with a further dose of VB-111. They showed SD for 8 months further. VB-111 was found to be safe and well tolerated by all study participants. The most frequent adverse events were mild (pyrexia, fatigue, chills) and no significant treatment-related adverse events observed.

Subsequently, a Phase II, nonrandomized clinical trial NCT01229865 [100] assessing the safety and efficacy of VB-111 in 29 patients with advanced DTC was completed in 2016. The trial is yet to be published and no results have been posted to date.

#### Combination therapy

Zhu *et al.* [101] conducted a randomized clinical study considering the efficacy of adjuvant administration of rAd-p53, a recombinant adenovirus encoding a human *p53* gene, combined with radiotherapy for the treatment of stage IV PTC in 2012. The results were presented by poster at the 2012 American Society of Oncology meeting. However, they have not been published formally. Although stage IV PTC is usually considered nonresectable, owing to the invasion of adjacent structures, Zhu *et al.* hypothesized that *rAd-P53* gene therapy would inhibit angiogenesis and increase radiosensitivity, thereby promoting resectability. Patients were randomized to receive either intratumoral rAd-p53 viral particles and radiotherapy (n = 25), or radiotherapy alone (n = 21). Patients who received rAd-p53 and radiotherapy had a resectability rate of 76% compared with 47.6% of patients who received radiotherapy alone. All of the 24% of patients who received rAd-p53 therapy and did not have resectable disease showed a PR, compared with 72% of the patients who received radiotherapy alone.

A large multicenter, randomized controlled Phase IV study considering the efficacy of recombinant rAd-p53 therapy with RAI, or in combination with surgery in patients with advanced TC commenced in 2009 (NCT00902122 [102]). However, no results have been reported and the last updates were posted in 2012.

## Resensitization therapy

Although DTC usually responds to RAI initially, they can become refractory to treatment. Radioiodine uptake is mediated by the *trans*-membrane sodium iodide symporter (NIS). Tumors can become refractory to treatment by suppressing NIS expression [103]. Studies have demonstrated DTC expressing the *BRAF*(V600E) mutation demonstrate suppressed NIS expression [104,105]. As such there is increasing attention to RAI resensitization therapies.

### Dabrafenib

Rothenberg *et al.* [106] conducted a Phase II trial studying the effectiveness of the selective *BRAF* inhibitor dabrafenib in promoting RAI resensitization in ten patients with BRAF-V600E-mutated RAI refractory PTC. Of these ten patients, six demonstrated restoration of iodine uptake and could be retreated with radioactive iodine. Responses were assessed 6 months following radioiodine treatment; five of the six patients demonstrated a reduction in the size of the target lesions on computed tomography imaging (two met the criteria for PR and four showed SD). Grade 1 and 2 treatment-related adverse effects (new cutaneous lesions, fatigue, gastrointestinal upset, electrolyte disturbance) were observed in up to 80% of patients. However, there were no serious treatment-related adverse events, and no dose reductions or discontinuations.

### Vemurafenib

Dunn *et al.* [31] investigated the effectiveness of the *BRAF* inhibitor vemurafenib in restoring radioiodine uptake in ten patients with RAI-refractory* BRAF*(V600E) mutant FTC. Six patients demonstrated increased RAI avidity, of whom four met the threshold for further RAI treatment. All four showed PFS at 6 months (two showed PR and two showed SD). Conversely, three of the four patients who did not show increased RAI uptake demonstrated further tumor growth. Grade 1–3 treatment-related adverse effects (rash, fatigue, palmar–plantar erythrodysesthesia, nausea) were seen in up to 90% of patients. However, there were no serious treatment-related adverse events, or discontinuations. Treatment was temporarily held in two patients, one for a grade III rash requiring a dose alteration, and another for an unrelated adverse event.

### Selumetinib

Ho *et al.* [29] investigated the effectiveness the MAPK kinase (MEK) 1 and MEK2 inhibitors in increasing I-124 uptake in 20 patients with FTC. Eight patients met the dosimetry threshold for further RAI treatment, of whom five showed PR and three showed SD. No grade 3 or higher treatment-related adverse effects were observed. More recently, the effectiveness of selumetinib followed by I-131 therapy in participants with RAI-refractory DTC was investigated in a UK single-arm Phase II clinical trial (SEL-I-METRY [107,108]). Recruitment ended in 2019 and results are yet to be published.

## Conclusion

The frequent progression of DTC to treatment refractory disease and the poor outcomes associated promote the need for effective novel treatment modalities. Although there have been some successes demonstrated with TKI treatment, their efficacy is short lived and treatment-associated adverse outcomes are common [18,19]. While Dadu *et al.* [62] and Cabanillas *et al.* [30] have demonstrated optimistic treatment options with second- and third-line alternatives for a proportion of patients who developed treatment failure with first-line TKI therapy, both studies had low participant numbers (60 and 25, respectively) and further larger-scale Phase III trials are needed.

Although both of the two Phase III clinical trials considering TKI in DTC treatment demonstrated positive outcomes [20,21], the relative efficacy of each treatment could not be established as the trials were not comparable. Phase III trials that compare multiple TKI, rather than a single drug and a placebo are required to address this question. To date, there is no research that elucidates the causative mechanisms underpinning variable treatment response despite numerous pathways having been identified; treatment failure with one particular TKI may indicate targeting of a pathway that is unrelated to carcinogenesis in any particular patient. Identifying the active pathways involved in an individual patient’s thyroid cancer may allow for a more tailored and efficacious approach, albeit with significant resource implications. Alternatively, MKIs which target several pathways are likely to have greater efficacy than TKI that target a single pathway and there is no evidence to suggest that MKIs cause greater side effects compared with TKI.

Immunotherapy has revolutionized the treatment of many different cancers. Although some preclinical studies have demonstrated promising results in DTC, further clinical research is warranted.

Preclinical studies have demonstrated that multimodal immunotherapy may work synergistically in thyroid cancer treatment [78]. Although clinical trials investigating the safety and efficacy of mono-immunotherapy are needed first and foremost, it may well be that trials considering multimodal therapy including an immunotherapy arm follow.

This review has considered the various secondary treatments for advanced RAI refractory DTC. Outcomes remain relatively poor, and there continues to be a significant gap of evidence considering the relative merits of the secondary treatments currently available. Efforts to understand the biological mechanisms underpinning treatment efficacy and the use of combined, individualized therapy offer exciting prospects for future research and patient outcomes.

## Future perspective

The current trend of increasing thyroid cancer incidence predicts an increased burden of disease over the next 5–10 years. Although it is anticipated that surgical excision with the addition of the RAI for more advanced disease will continue to be the mainstay of thyroid cancer treatment, it is expected that the use of secondary therapies including novel mono or multiple TKI therapy and/or immunotherapy will become more routine.

It is highly likely that other TKIs, in addition to sorafenib and levantinib, will gain FDA approval following the conclusion of ongoing and future clinical trials (VERIFY, NCT01876784, COSMIC-311, NCT03690388, NCT03048877). Given the limited duration of TKI efficacy, approved second- and third-line options for patients with DTC who experience first-line treatment failure will be beneficial. Although small clinical studies investigating the efficacy of alternative TKIs following first-line treatment failure have demonstrated positive results [30,62], larger studies are needed.

While immunotherapy has become established as an effective treatment modality for various cancers in recent years, published research regarding its efficacy in DTC is limited. Results from small clinical trials [80] have shown promise and the results of larger Phase II clinical trials are eagerly awaited (KEYNOTE-28, KEYNOTE-158, DUTHY, NCT03753919).

The role of gene-targeted therapy is playing an increasing role in the treatment of many cancers with specific genetic mutations. Positive results seen in large clinical trials involving patients with solid tumors [28] have, to date, been mirrored in smaller trials involving patients with thyroid cancer [29–31]. It is therefore anticipated that large-scale clinical trials evaluating the efficacy of targeted treatment for patients with DTC harboring specific gene mutations will follow.

The use of multimodal therapy in thyroid cancer management has demonstrated positive outcomes in preclinical studies [78]. While it is likely that the efficacy of multimodal therapy will continue to be explored, it is anticipated that it may be some time before its routine clinical use is established.

**Executive summary**

- Thyroid cancer incidence is increasing.
- Secondary treatment options are needed for patients with treatment refractory disease.
- Twenty-three published studies and six ongoing clinical trials were identified in a systematic review of secondary treatment options for advanced/treatment refractory differentiated thyroid cancer (DTC).
- Two tyrosine kinase inhibitors (TKIs), sorafenib and levantininb, have received the US FDA approval for DTC treatment.
- Several alternative TKIs have shown promising results in clinical trials, but are currently not FDA approved: vandetanib, cabozantinib, apatinib, vemurafenib, pazopanib, sunitinib, larotrectinib.
- Combination TKI therapy (sorafenib and temsirolimus) has shown promise in a preliminary study.
- Treatment failure with first-line TKI is common. Salvage TKI therapy has been shown to be efficacious following treatment failure in small studies.
- The immune checkpoint inhibitor pembrolizumab was demonstrated to be effective in treating patients with advanced DTC in a Phase Ib trial (KEYNOTE-28).
- Immune checkpoint inhibitor efficacy in DTC is currently under investigation in two ongoing clinical trials (DUTHY and KEYNOTE-158).
- *In vitro*, chimeric antigen receptor T cells have displayed ‘robust and specific killing’ against DTC cell lines.
- *In vitro*, the *BRAF* inhibitor PLX4720 reduced *NY-ESO-1* gene expression in some *BRAF* mutant thyroid cancer cell lines.
- *In vitro*, the third-generation herpes simplex virus vector, G47Δ, has been shown to kill DTC cells.
- The efficacy of the engineered antiangiogenic adenovirus VB-111 intreating patients with advanced DTC was studied in a Phase II clinical trial, which was completed in 2016.
- Resensitizing agents that promote radioactive iodine ablation reuptake have shown promising results in preliminary clinical trials (Vemurafenib, Selumetinib) and continue to be evaluated in a Phase II trial (SEL-I-METRY).
